# Synthesis and crystal structure of di­bromido­bis­[1-butyl-4-(pyridin-2-yl)-1,2,4-triazol-5-yl­idene]palladium(II)

**DOI:** 10.1107/S2056989026009928

**Published:** 2026-10-02

**Authors:** Anes Kovacevic, Sean Parkin

**Affiliations:** a Department of Chemistry Berea College,Berea KY 40404 USA; b Department of Chemistry University of Kentucky,Lexington KY 40506-0055 USA; Institute of Chemistry, Chinese Academy of Sciences

**Keywords:** crystal structure, synthesis, disorder

## Abstract

The synthesis and structure of di­bromido­bis­[1-butyl-4-(pyridin-2-yl)-1,2,4-triazol-5-yl­idene]palladium(II), [PdBr_2_(C_11_H_14_N_4_)_2_] (**I**), a triazolium-containing N-heterocyclic carbene (NHC), are presented. The compound was prepared by the direct base-free reaction of 1-butyl-4-(pyridin-2-yl)-1,2,4-triazolium bromide with Pd_2_(dba)_3_. The title compound crystallizes with the symmetry of space group *P*2_1_/*c*, with the Pd^II^ atom on a centre of inversion, adopting a square-planar *trans* geometry. The structure is extensively disordered as a result of a ∼180° flip of the triazolium ligands, which effectively superimposes disordered *n*-butyl and pyridyl components. In the crystal, supra­molecular inter­actions include pairs of inter­molecular C—H⋯N weak hydrogen bonds between inversion-related pyridine rings [*R*^2^_2_(6) motifs] to form mono-periodic chains parallel to [101]. There is also π-stacking of inversion-related triazole rings, which propagate to form chains in the [100] direction.

## Abstract 2

The synthesis and crystal structure of di­bromido­bis­[1-butyl-4-(pyridin-2-yl)-1,2,4-triazol-5-yl­idene]palladium(II) are presented

## 1. Chemical context

N-Heterocyclic carbenes (NHCs) have become important ligands in organometallic chemistry since the isolation of the first stable crystalline carbene by Arduengo and co-workers (Arduengo *et al.*, 1991 ▸). Their strong σ-donor properties, readily tunable steric and electronic characteristics, and diverse coordination modes have led to extensive applications in transition-metal chemistry and catalysis (Crabtree, 2005 ▸; Fortman & Nolan, 2011 ▸; Díez-González *et al.*, 2009 ▸). Both normal and abnormal coordination modes of NHCs have been investigated, with abnormal C5-bound NHCs shown to be particularly strong electron-donor ligands (Chianese *et al.*, 2004 ▸). Palladium-NHC complexes have been particularly well studied because of their importance in homogeneous catalysis and coordination chemistry (Crabtree, 2005 ▸; Fortman & Nolan, 2011 ▸). Although imidazolium-derived NHC systems are well established, the coordination chemistry of triazolium-derived NHCs has received comparatively less attention (Kovacevic, Faller *et al.*, 2025 ▸; Kovacevic, Parkin *et al.*, 2025 ▸).

Recent studies have demonstrated that triazolium salts can exhibit both normal and abnormal coordination modes toward transition-metal centers. In particular, pyridyl-substituted triazolium ligands have been shown to display counter-ion-dependent coordination behavior at iridium (Kovacevic, Faller *et al.*, 2025 ▸). More recently, 1,2,4-triazolium salts were shown to undergo direct C—H oxidative addition with Pd_2_(dba)_3_ under base-free conditions to give palladium(II) bis­(NHC) complexes, which were isolated predominantly as *trans-anti* and *trans-syn* rotamers together with minor *cis* products (Kovacevic, Parkin *et al.*, 2025 ▸).

As part of ongoing studies of the structural and electronic factors governing this class of palladium complexes, a pyridine-substituted triazolium salt was examined because the pyridyl group provides an additional potential coordination site. The title compound, bearing butyl and pyridyl substituents, was prepared by direct reaction of the triazolium salt with Pd_2_(dba)_3_ and crystallizes as the *trans* bis­(NHC)palladium(II) isomer, **I**.
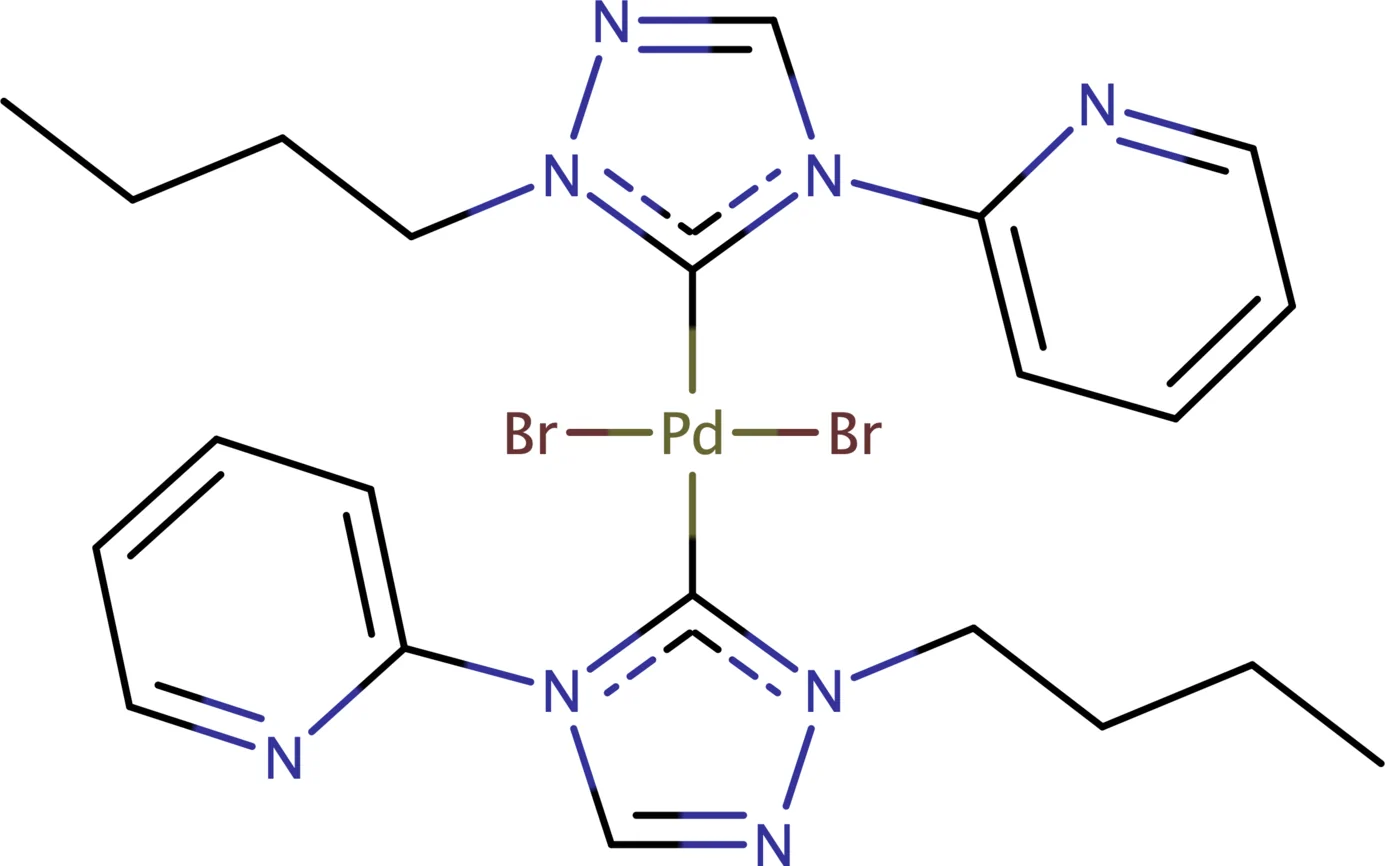


## 2. Structural commentary

**Figure 1:**
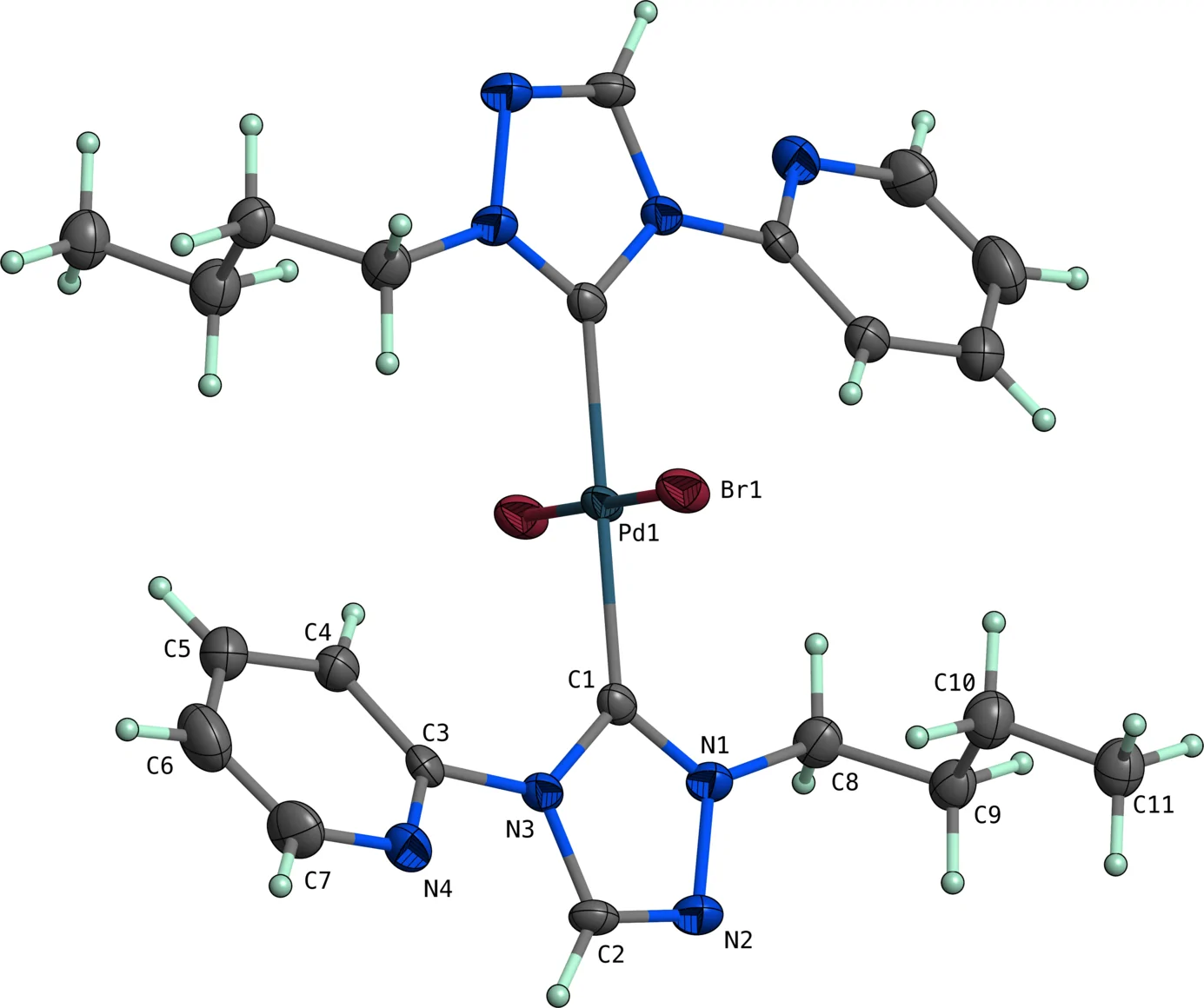
An ellipsoid plot of **I** (50% probability) showing the mol­ecular conformation of the major disorder component. Hydrogen atoms are drawn as small arbitrary spheres.

**Figure 2:**
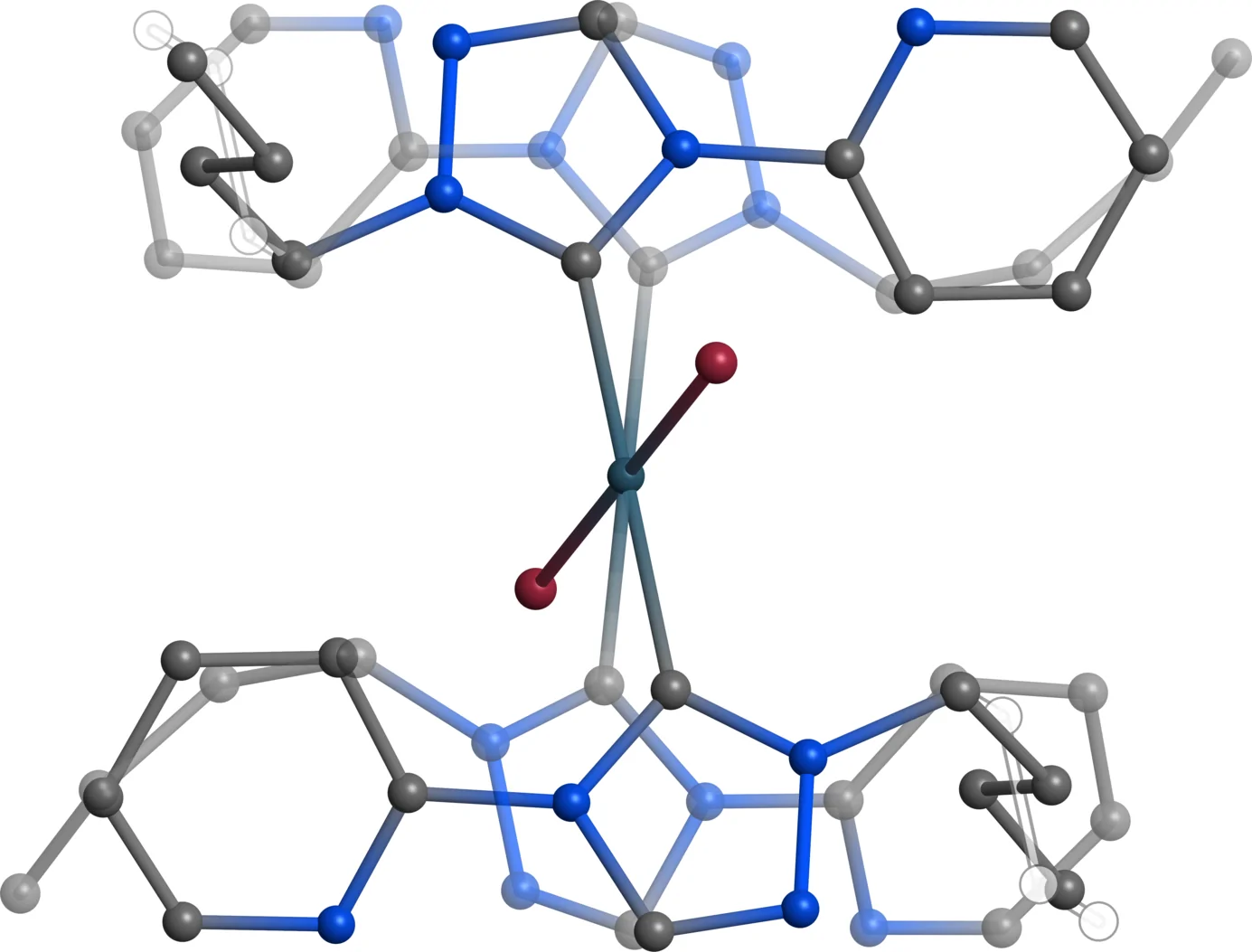
The nature of disorder in **I**. The major conformer is shown as a solid opaque ball-and-stick view, while the minor components are drawn with semi-transparent colours. Additional sub-disorder of the *n*-butyl group is shown with open semi-transparent atoms/bonds.

The structure of **I** is centrosymmetric, space group *P*2_1_/*c*, with *Z*′ = 0.5. In the chosen asymmetric unit, the Pd atom sits at (0.5, 0.5, 0.5) and is connected to Br1, Br1^i^, and C1, C1^i^ [symmetry code: (i) 1 − *x*, 1 − *y*, 1 − *z*] atoms in a square-planar *trans* arrangement (Fig. 1 ▸). Although the PdBr_2_ moiety is not disordered, the overall structure is extensively disordered, requiring modelling of four disorder component occupancies. The whole of the 1-butyl-4-pyridyl-1,2,4-triazolium ligand is disordered by an approximate 180° flip, effectively superimposing the *n*-butyl and pyridyl groups, and there is further disorder of one of the *n*-butyl components (Fig. 2 ▸). This complex disorder is handled with the PART, FVAR, and SUMP commands in *SHELXL* (Sheldrick, 2015*b* ▸) giving occupancy factors of 0.9251 (14) and 0.0749 (14) for the main ‘flip’, with the major *n*-butyl component being further disordered over two sites with occupancies of 0.786 (2) and 0.138 (2). Given the low occupancies of the minor parts, the following description is restricted to the major components. The dihedral angle between the central PdBr_2_C_2_ core and the triazole is 72.99 (8)°, while that between the triazole and pyridyl rings is 33.25 (18)°.

## 3. Supra­molecular features

**Figure 3:**
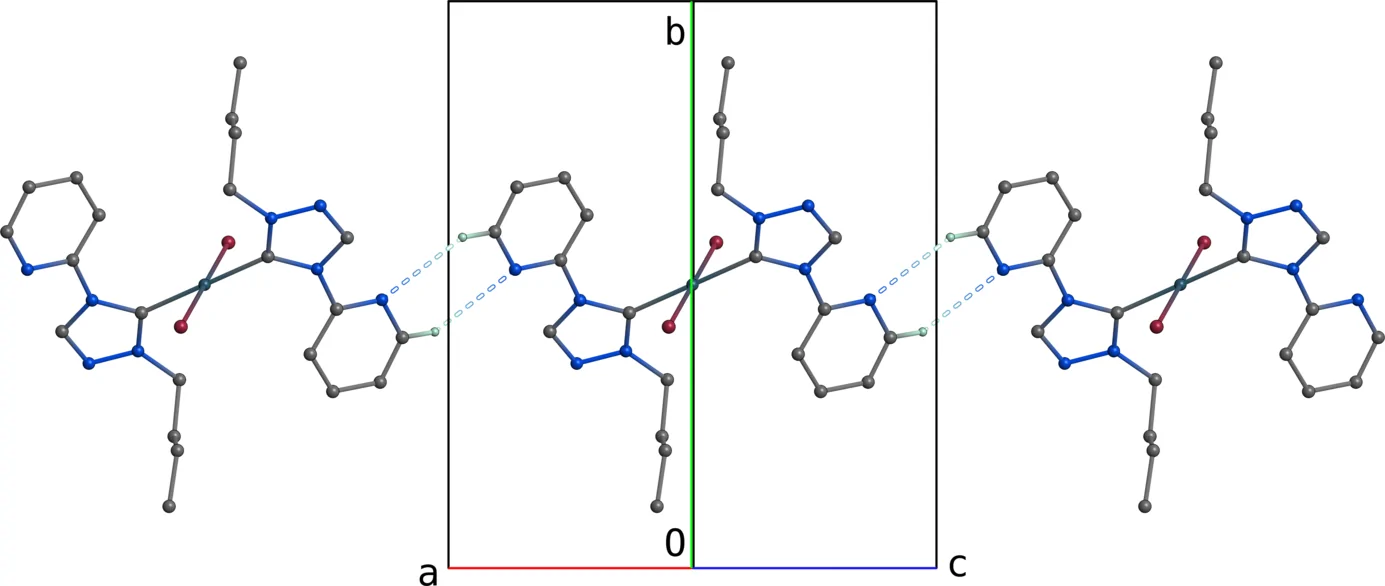
Weak C—H⋯N4 hydrogen-bonded pairs form 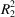
(6) ring motifs that join the mol­ecules into chains parallel to [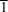
01]. Hydrogen atoms not involved in the inter­action are suppressed.

There are no conventional hydrogen bonds in the structure of **I**. The geometric criteria in *PrimeXP* (https://xray.uky.edu/Resources/primeXP.html; Parkin, 2026 ▸) suggest several C—H⋯Br contacts, which all stem from the Br1 atom residing in a pocket surrounded by disordered hydrogen atoms. Because these contacts involve low-occupancy hydrogens, they presumably lack the structural weight to exert major influence on the crystal packing. The inter­actions with the major component, C2—H2⋯Br1^iii^ [symmetry code: (iii) *x* + 1, *y*, *z*] and C5—H5⋯Br1^iv^ [symmetry code: (iv) −*x* + 1, 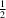 + *y*, 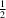 − *z*], are listed in Table 1 ▸, though structurally would be rather weak. There are also mutual pairs of C7—H7⋯N4^ii^ [symmetry code: (ii) 2 − *x*, 1 − *y*, −*z*] inter­actions between inversion-related pyridine rings in an 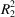
(6) motif (Etter *et al.*, 1990 ▸), which combine to link mol­ecules parallel to the [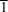
01] direction, as shown in Fig. 3 ▸.

**Figure 4:**
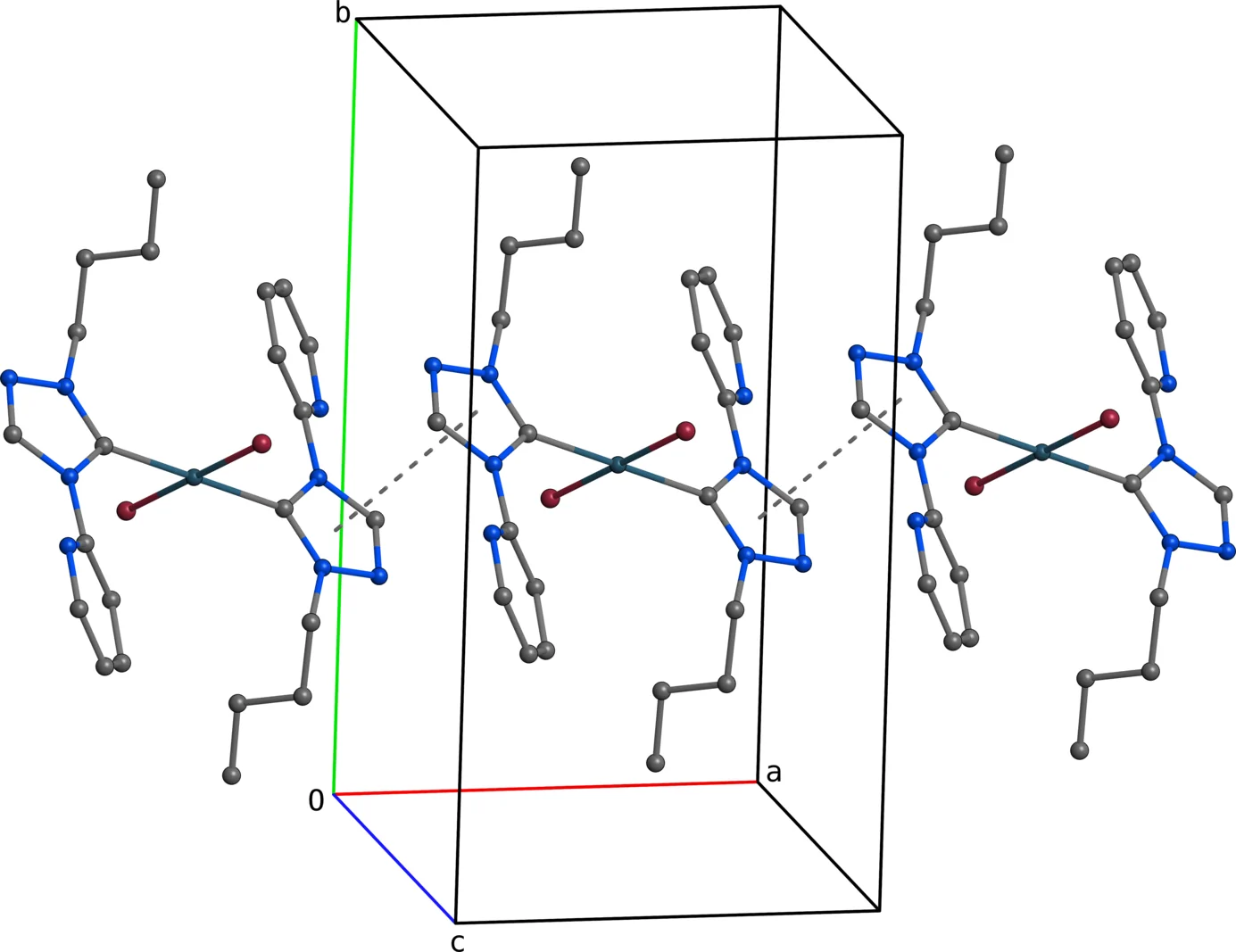
The triazole rings of inversion-related adjacent mol­ecules of **I** are π-stacked, forming chains that propagate parallel to the crystallographic *a* axis

In addition to the weak hydrogen-bond constructs, the triazole rings are π-stacked with inversion-related (2 − *x*, 1 − *y*, 1 − *z*) copies to form chains that propagate along the *a*-axis direction (Fig. 4 ▸). In these pairs, the inter­planar ring separation is 3.371 (5) Å and the ring centroid-centroid distance is 3.621 (5) Å.

## 4. Database survey

A search of the Cambridge Structural Database (CSD v2026.1.1 with updates through May 2026; Groom *et al.*, 2016) using a search fragment consisting of a Pd atom with two ‘any’ ligands and two 1,2,4-triazole rings attached by carbon returned 48 hits. Changing the two ‘any’ ligands to ‘any halogen’, gave 35 hits, while specifying these as bromine gave 25 matches. Of these 25, three had little in common with **I**. Of the remaining 22 structures, eight had an aromatic ring substituent attached directly to a nitro­gen adjacent to the carbon bonded to Pd. These were CIKBOQ and CIKCAD, both with phenyl groups (Nguyen *et al.*, 2018 ▸); JAXCUJ, JAXDEU (mesit­yl) and JAXDAQ, JAXDIY (2,6-diiso­propyl­phen­yl) (Nguyen *et al.*, 2017 ▸); REYKEP (mesit­yl) and REYKIT (2,6-diiso­propyl­phen­yl) (Nguyen *et al.*, 2022 ▸). The two remaining entries, UMAPIL and UMATEL (Kovacevic, Parkin *et al.*, 2025 ▸), from our own laboratories, are *cis* and *trans* isomers, each having benzyl and *n*-butyl groups attached to their triazole rings. The *trans* isomer, UMATEL, has the most in common with **I**.

## 5. Synthesis and crystallization

The palladium complex was prepared by a procedure analogous to that reported previously for related palladium(II) bis­(NHC) complexes derived from 1,2,4-triazolium salts (Kovacevic, Parkin *et al.*, 2025 ▸). A mixture of 1-butyl-4-(pyridin-2-yl)-1,2,4-triazolium bromide (74 mg, 0.26 mmol) and Pd_2_(dba)_3_ (62 mg, 0.068 mmol) was refluxed in anhydrous THF (5 mL) for 3 h. After cooling, THF was removed under reduced pressure. The residue was dissolved in a minimal amount of di­chloro­methane and filtered through celite. Upon addition of diethyl ether, the palladium complex precipitated and was isolated (37 mg, 43%). Crystals suitable for X-ray diffraction were obtained from a di­chloro­methane/diethyl ether solution.

^1^H NMR (acetone-*d*_6_, 298 K): δ 9.69 (*d*, 2H, H_pyr_), 9.62 (*s*, 2H, H_trz_), 8.46 (*t*, 2H, H_pyr_), 8.32 (*d*, 2H, H_pyr_), 7.73 (*m*, 2H, H_pyr_), 5.00 (*t*, 4H, H_CH2-Bu_), 1.91 (*m*, 4H, H_CH2-Bu_), 1.44 (*m*, 4H, H_CH2-Bu_), 0.96 (*t*, 6H, H_CH3-Bu_).

## 6. Refinement

Crystal data, data collection and structure refinement details are summarized in Table 2 ▸. H atoms were found in difference-Fourier maps, but subsequently included in the refinement using riding models, with constrained distances set to 0.95 Å (C*sp*^2^—H), 0.98 Å (*R*CH_3_) and 0.99 Å (*R*_2_CH_2_). *U*_iso_(H) parameters were set to values of either 1.2*U*_eq_ or 1.5*U*_eq_ (*R*CH_3_ only) of the attached atom.

To ensure satisfactory refinement for disordered groups in the structure, a combination of constraints and restraints were employed. The constraints (*SHELXL* commands EXYZ and EADP) were used to fix overlapping fragments. Restraints were used to ensure the integrity of ill-defined or disordered groups (*SHELXL* commands SAME, SADI, FLAT, SIMU, RIGU, and SUMP).

## Supplementary Material

Crystal structure: contains datablock(s) I, global. DOI: 10.1107/S2056989026009928/nx2039sup1.cif

Structure factors: contains datablock(s) I. DOI: 10.1107/S2056989026009928/nx2039Isup2.hkl

CCDC reference: 2590397

Additional supporting information:  crystallographic information; 3D view; checkCIF report

## Figures and Tables

**Table 1:** Weak hydrogen bonds (Å, °) in **I**

*D*—H⋯*A*	*D*—H	H⋯*A*	*D*⋯*A*	*D*—H⋯*A*
C7—H7—N4^ii^	0.95	2.73	3.574 (4)	149.0
C2—H2⋯Br1^iii^	0.95	2.82	3.653 (3)	147.6
C5—H5⋯Br1^iv^	0.95	2.84	3.747 (3)	158.9

Symmetry codes: (ii) 2 − *x*, 1 − *y*, −*z*; (iii) *x* + 1, *y*, *z*; (i)= −*x* + 1, 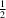 + *y*, 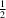 − *z*.

**Table 2:** Experimental details

Crystal data	
Chemical formula	[PdBr_2_(C_11_H_14_N_4_)_2_]
*M*_r_	670.74
Crystal system, space group	Monoclinic, *P*2_1_/*c*
Temperature (K)	100
*a*, *b*, *c* (Å)	8.1911 (2), 14.9855 (3), 10.2521 (3)
β (°)	90.730 (1)
*V* (Å^3^)	1258.32 (5)
*Z*	2
Radiation type	Mo *K*α
μ (mm^−1^)	3.94
Crystal size (mm)	0.12 × 0.12 × 0.02

Data collection	
Diffractometer	Bruker D8 Venture dual source
Absorption correction	Multi-scan (*SADABS*; Krause *et al.*, 2015 ▸)
*T*_min_, *T*_max_	0.734, 0.862
No. of measured, independent and observed [*I* > 2σ(*I*)] reflections	22432, 3131, 2541
*R*_int_	0.036
(sin θ/λ)_max_ (Å^−1^)	0.667

Refinement	
*R*[*F*^2^ > 2σ(*F*^2^)], *wR*(*F*^2^), *S*	0.026, 0.047, 1.04
No. of reflections	3131
No. of parameters	312
No. of restraints	892
H-atom treatment	H-atom parameters constrained
Δρ_max_, Δρ_min_ (e Å^−3^)	0.56, −0.50

Computer programs: *APEX5* (Bruker, 2023 ▸), *SHELXT* (Sheldrick, 2015*a* ▸), *SHELXL2025/1* (Sheldrick, 2015*b* ▸), *PrimeXP* (Parkin, 2026 ▸), *SHELX* (Sheldrick, 2008 ▸), *PLATON* (Spek, 2020 ▸) and *publCIF* (Westrip, 2010 ▸).

## supplementary crystallographic information

### Dibromidobis[1-butyl-4-(pyridin-2-yl)-1,2,4-triazol-5-ylidene]palladium(II) Crystal data

**Table:**

[PdBr_2_(C_11_H_14_N_4_)_2_]	*F*(000) = 664
*M**_r_* = 670.74	*D*_x_ = 1.770 Mg m^−^^3^
Monoclinic, *P*2_1_/*c*	Mo *K*α radiation, λ = 0.71073 Å
*a* = 8.1911 (2) Å	Cell parameters from 9922 reflections
*b* = 14.9855 (3) Å	θ = 2.5–28.3°
*c* = 10.2521 (3) Å	µ = 3.94 mm^−^^1^
β = 90.730 (1)°	*T* = 100 K
*V* = 1258.32 (5) Å^3^	Plate, yellow
*Z* = 2	0.12 × 0.12 × 0.02 mm

### Dibromidobis[1-butyl-4-(pyridin-2-yl)-1,2,4-triazol-5-ylidene]palladium(II) Data collection

**Table:**

Bruker D8 Venture dual source diffractometer	3131 independent reflections
Radiation source: microsource	2541 reflections with *I* > 2σ(*I*)
Detector resolution: 7.41 pixels mm^-1^	*R*_int_ = 0.036
φ and ω scans	θ_max_ = 28.3°, θ_min_ = 2.5°
Absorption correction: multi-scan (*SADABS*; Krause *et al*., 2015)	*h* = −10→10
*T*_min_ = 0.734, *T*_max_ = 0.862	*k* = −18→20
22432 measured reflections	*l* = −13→13

### Dibromidobis[1-butyl-4-(pyridin-2-yl)-1,2,4-triazol-5-ylidene]palladium(II) Refinement

**Table:**

Refinement on *F*^2^	Secondary atom site location: difference Fourier map
Least-squares matrix: full	Hydrogen site location: difference Fourier map
*R*[*F*^2^ > 2σ(*F*^2^)] = 0.026	H-atom parameters constrained
*wR*(*F*^2^) = 0.047	*w* = 1/[σ^2^(*F*_o_^2^) + (0.0153*P*)^2^ + 0.1867*P*] where *P* = (*F*_o_^2^ + 2*F*_c_^2^)/3
*S* = 1.04	(Δ/σ)_max_ < 0.001
3131 reflections	Δρ_max_ = 0.56 e Å^−^^3^
312 parameters	Δρ_min_ = −0.50 e Å^−^^3^
892 restraints	Extinction correction: SHELXL2025/1 (Sheldrick 2015b), Fc^*^=kFc[1+0.001xFc^2^λ^3^/sin(2θ)]^-1/4^
Primary atom site location: structure-invariant direct methods	Extinction coefficient: 0.0011 (3)

### Dibromidobis[1-butyl-4-(pyridin-2-yl)-1,2,4-triazol-5-ylidene]palladium(II) Special details

**Table:**

Experimental. The crystal was mounted using polyisobutene oil on the tip of a fine glass fibre, which was fastened in a copper mounting pin with electrical solder. It was placed directly into the cold gas stream of a liquid-nitrogen based cryostat (Hope, 1994; Parkin & Hope, 1998). Diffraction data were collected with the crystal at 100K.
Geometry. All esds (except the esd in the dihedral angle between two l.s. planes) are estimated using the full covariance matrix. The cell esds are taken into account individually in the estimation of esds in distances, angles and torsion angles; correlations between esds in cell parameters are only used when they are defined by crystal symmetry. An approximate (isotropic) treatment of cell esds is used for estimating esds involving l.s. planes.
Refinement. Refinement progress was checked using *Platon* (Spek, 2020) and by an *R*-tensor (Parkin, 2000). The final model was further checked with the IUCr utility *checkCIF*.

### Dibromidobis[1-butyl-4-(pyridin-2-yl)-1,2,4-triazol-5-ylidene]palladium(II) Fractional atomic coordinates and isotropic or equivalent isotropic displacement parameters (Å^2^)

**Table:**

	*x*	*y*	*z*	*U*_iso_*/*U*_eq_	Occ. (<1)
Pd1	0.500000	0.500000	0.500000	0.02105 (8)	
Br1	0.40050 (3)	0.42570 (2)	0.30425 (3)	0.02899 (9)	
C1	0.7252 (3)	0.45029 (16)	0.4652 (3)	0.0203 (6)	0.9251 (14)
N1	0.8016 (2)	0.38293 (13)	0.5270 (2)	0.0239 (5)	0.786 (2)
N2	0.9510 (2)	0.36100 (14)	0.4750 (2)	0.0284 (5)	0.9251 (14)
C2	0.9663 (4)	0.4174 (2)	0.3803 (5)	0.0233 (10)	0.9251 (14)
H2	1.058284	0.420338	0.324947	0.028*	0.9251 (14)
N3	0.8323 (2)	0.47247 (13)	0.3701 (2)	0.0209 (5)	0.9251 (14)
C3	0.8187 (3)	0.54327 (16)	0.2772 (3)	0.0231 (6)	0.9251 (14)
C4	0.7433 (3)	0.62269 (17)	0.3100 (3)	0.0281 (7)	0.9251 (14)
H4	0.694942	0.631301	0.392760	0.034*	0.9251 (14)
C5	0.7418 (4)	0.6885 (2)	0.2165 (3)	0.0373 (7)	0.9251 (14)
H5	0.690636	0.744185	0.232663	0.045*	0.9251 (14)
C6	0.8168 (9)	0.6720 (5)	0.0977 (4)	0.0474 (10)	0.9251 (14)
H6	0.817328	0.716481	0.031685	0.057*	0.9251 (14)
C7	0.8877 (4)	0.59314 (19)	0.0773 (3)	0.0374 (7)	0.9251 (14)
H7	0.938351	0.583436	−0.004319	0.045*	0.9251 (14)
N4	0.8919 (3)	0.52663 (14)	0.1652 (2)	0.0297 (6)	0.9251 (14)
C8	0.7448 (4)	0.33224 (19)	0.6399 (3)	0.0289 (7)	0.786 (2)
H8A	0.634528	0.353285	0.663740	0.035*	0.786 (2)
H8B	0.819062	0.343306	0.715075	0.035*	0.786 (2)
C9	0.7384 (4)	0.2323 (2)	0.6120 (3)	0.0287 (7)	0.786 (2)
H9A	0.848445	0.211937	0.586546	0.034*	0.786 (2)
H9B	0.708953	0.200518	0.692950	0.034*	0.786 (2)
C10	0.6175 (4)	0.2074 (2)	0.5053 (3)	0.0342 (8)	0.786 (2)
H10A	0.642979	0.242014	0.425849	0.041*	0.786 (2)
H10B	0.506567	0.224609	0.533056	0.041*	0.786 (2)
C11	0.6178 (5)	0.1089 (2)	0.4718 (5)	0.0336 (10)	0.786 (2)
H11A	0.727677	0.090946	0.445635	0.050*	0.786 (2)
H11B	0.540608	0.097791	0.399785	0.050*	0.786 (2)
H11C	0.585286	0.074257	0.548257	0.050*	0.786 (2)
C1'	0.726 (3)	0.4823 (14)	0.431 (4)	0.021 (2)	0.0749 (14)
N1'	0.824 (2)	0.5404 (12)	0.369 (3)	0.022 (2)	0.0749 (14)
N2'	0.974 (2)	0.5043 (13)	0.341 (3)	0.022 (2)	0.0749 (14)
C2'	0.950 (6)	0.4196 (18)	0.362 (7)	0.023 (2)	0.0749 (14)
H2'	1.016079	0.373051	0.328939	0.028*	0.0749 (14)
N3'	0.814 (3)	0.4066 (11)	0.438 (3)	0.0218 (19)	0.0749 (14)
C3'	0.764 (3)	0.3245 (12)	0.493 (2)	0.0252 (18)	0.0749 (14)
C4'	0.699 (3)	0.3216 (18)	0.617 (2)	0.027 (2)	0.0749 (14)
H4'	0.678681	0.375064	0.664320	0.033*	0.0749 (14)
C5'	0.664 (4)	0.2403 (16)	0.671 (3)	0.030 (2)	0.0749 (14)
H5'	0.620697	0.235038	0.756168	0.036*	0.0749 (14)
C6'	0.696 (4)	0.1660 (16)	0.595 (3)	0.031 (2)	0.0749 (14)
H6'	0.673999	0.108030	0.627854	0.038*	0.0749 (14)
C7'	0.758 (4)	0.1750 (15)	0.476 (3)	0.031 (2)	0.0749 (14)
H7'	0.771352	0.122569	0.424910	0.037*	0.0749 (14)
N4'	0.802 (3)	0.2536 (12)	0.423 (2)	0.029 (3)	0.0749 (14)
C8'	0.787 (5)	0.6339 (15)	0.342 (3)	0.027 (2)	0.0749 (14)
H8'1	0.888076	0.669626	0.349784	0.033*	0.0749 (14)
H8'2	0.709034	0.656259	0.407889	0.033*	0.0749 (14)
C9'	0.713 (4)	0.645 (2)	0.205 (3)	0.032 (2)	0.0749 (14)
H9'1	0.674704	0.586046	0.173898	0.039*	0.0749 (14)
H9'2	0.615838	0.684394	0.211027	0.039*	0.0749 (14)
C10'	0.828 (10)	0.684 (6)	0.107 (3)	0.037 (3)	0.0749 (14)
H10C	0.941426	0.672849	0.138950	0.045*	0.0749 (14)
H10D	0.811869	0.749012	0.104364	0.045*	0.0749 (14)
C11'	0.813 (4)	0.648 (2)	−0.031 (3)	0.040 (5)	0.0749 (14)
H11D	0.893973	0.677265	−0.085904	0.060*	0.0749 (14)
H11E	0.833350	0.583340	−0.030197	0.060*	0.0749 (14)
H11F	0.703383	0.659743	−0.064893	0.060*	0.0749 (14)
N1"	0.8016 (2)	0.38293 (13)	0.5270 (2)	0.0239 (5)	0.139 (2)
C8"	0.7448 (4)	0.33224 (19)	0.6399 (3)	0.0289 (7)	0.139 (2)
H8"1	0.677966	0.371021	0.696345	0.035*	0.139 (2)
H8"2	0.839396	0.310427	0.691764	0.035*	0.139 (2)
C9"	0.642 (2)	0.2524 (9)	0.5907 (19)	0.0294 (18)	0.139 (2)
H9"1	0.582020	0.225721	0.664508	0.035*	0.139 (2)
H9"2	0.560161	0.273391	0.525372	0.035*	0.139 (2)
C10"	0.751 (2)	0.1828 (10)	0.530 (2)	0.032 (2)	0.139 (2)
H10E	0.801142	0.209047	0.451412	0.038*	0.139 (2)
H10F	0.839796	0.168514	0.592450	0.038*	0.139 (2)
C11"	0.667 (3)	0.0969 (14)	0.491 (3)	0.0336 (10)	0.139 (2)
H11G	0.747422	0.056273	0.452410	0.050*	0.139 (2)
H11H	0.619637	0.068946	0.567734	0.050*	0.139 (2)
H11I	0.580886	0.109580	0.426342	0.050*	0.139 (2)

### Dibromidobis[1-butyl-4-(pyridin-2-yl)-1,2,4-triazol-5-ylidene]palladium(II) Atomic displacement parameters (Å^2^)

**Table:**

	*U*^11^	*U*^22^	*U*^33^	*U*^12^	*U*^13^	*U*^23^
Pd1	0.01699 (13)	0.01911 (13)	0.02729 (17)	−0.00128 (10)	0.01001 (11)	−0.00499 (11)
Br1	0.02313 (14)	0.02974 (14)	0.03430 (18)	−0.00239 (10)	0.00832 (11)	−0.01150 (11)
C1	0.0206 (13)	0.0177 (13)	0.0229 (16)	−0.0023 (10)	0.0066 (11)	−0.0029 (10)
N1	0.0221 (11)	0.0236 (11)	0.0262 (13)	0.0033 (8)	0.0081 (10)	0.0004 (9)
N2	0.0222 (11)	0.0284 (11)	0.0348 (15)	0.0050 (9)	0.0086 (10)	0.0007 (10)
C2	0.0170 (14)	0.0272 (13)	0.026 (2)	0.0038 (11)	0.0062 (15)	−0.0013 (12)
N3	0.0191 (10)	0.019 (1)	0.0248 (13)	−0.0002 (8)	0.0072 (9)	−0.0025 (9)
C3	0.0176 (12)	0.0200 (12)	0.0317 (17)	−0.0053 (9)	0.0047 (12)	0.0022 (11)
C4	0.0264 (17)	0.0229 (13)	0.0353 (19)	0.0000 (11)	0.0087 (13)	0.0005 (12)
C5	0.0339 (16)	0.0275 (14)	0.051 (2)	0.0002 (13)	0.0063 (15)	0.0074 (14)
C6	0.046 (2)	0.045 (3)	0.052 (2)	−0.0111 (16)	0.0079 (18)	0.0163 (17)
C7	0.0389 (17)	0.0432 (17)	0.0303 (18)	0.0001 (13)	0.0119 (14)	0.0117 (13)
N4	0.0287 (12)	0.0284 (12)	0.0321 (15)	−0.0011 (9)	0.0115 (11)	0.0039 (10)
C8	0.0336 (16)	0.0274 (13)	0.0260 (16)	0.0032 (11)	0.0091 (12)	0.0033 (11)
C9	0.0330 (17)	0.0254 (15)	0.0279 (18)	0.0030 (13)	0.0073 (15)	0.0051 (13)
C10	0.0397 (18)	0.0292 (16)	0.034 (2)	0.0007 (14)	0.0038 (15)	0.0031 (14)
C11	0.041 (3)	0.0288 (17)	0.031 (2)	−0.0010 (16)	0.0031 (19)	0.0026 (15)
C1'	0.020 (4)	0.019 (4)	0.024 (4)	−0.002 (3)	0.007 (4)	−0.001 (4)
N1'	0.018 (4)	0.019 (3)	0.028 (4)	−0.003 (3)	0.008 (4)	−0.001 (4)
N2'	0.020 (4)	0.022 (4)	0.025 (4)	0.001 (3)	0.009 (4)	−0.003 (4)
C2'	0.020 (4)	0.024 (4)	0.026 (5)	0.001 (4)	0.008 (4)	−0.001 (4)
N3'	0.020 (3)	0.021 (3)	0.024 (4)	0.000 (3)	0.007 (3)	−0.002 (3)
C3'	0.026 (3)	0.024 (3)	0.026 (3)	0.002 (3)	0.008 (3)	0.003 (3)
C4'	0.029 (4)	0.027 (4)	0.026 (4)	0.002 (4)	0.009 (4)	0.004 (4)
C5'	0.035 (4)	0.027 (3)	0.029 (4)	0.002 (4)	0.008 (4)	0.004 (3)
C6'	0.037 (4)	0.027 (4)	0.031 (4)	0.000 (4)	0.008 (4)	0.003 (4)
C7'	0.037 (4)	0.026 (4)	0.029 (4)	0.000 (4)	0.007 (4)	0.002 (4)
N4'	0.033 (5)	0.025 (4)	0.030 (5)	0.001 (5)	0.010 (5)	0.002 (4)
C8'	0.024 (5)	0.020 (4)	0.038 (4)	−0.001 (4)	0.009 (4)	0.001 (4)
C9'	0.030 (4)	0.025 (4)	0.042 (4)	−0.003 (4)	0.006 (4)	0.005 (4)
C10'	0.036 (5)	0.034 (5)	0.043 (5)	−0.005 (5)	0.006 (5)	0.010 (5)
C11'	0.036 (10)	0.045 (10)	0.039 (7)	−0.013 (10)	0.006 (8)	0.012 (8)
N1"	0.0221 (11)	0.0236 (11)	0.0262 (13)	0.0033 (8)	0.0081 (10)	0.0004 (9)
C8"	0.0336 (16)	0.0274 (13)	0.0260 (16)	0.0032 (11)	0.0091 (12)	0.0033 (11)
C9"	0.033 (3)	0.027 (3)	0.028 (4)	0.002 (3)	0.009 (3)	0.004 (3)
C10"	0.038 (4)	0.027 (3)	0.030 (4)	0.001 (3)	0.009 (4)	0.004 (4)
C11"	0.041 (3)	0.0288 (17)	0.031 (2)	−0.0010 (16)	0.0031 (19)	0.0026 (15)

### Dibromidobis[1-butyl-4-(pyridin-2-yl)-1,2,4-triazol-5-ylidene]palladium(II) Geometric parameters (Å, º)

**Table:**

Pd1—C1'^i^	2.006 (15)	N2'—C2'	1.30 (2)
Pd1—C1'	2.006 (15)	C2'—N3'	1.381 (18)
Pd1—C1^i^	2.025 (2)	C2'—H2'	0.9500
Pd1—C1	2.025 (2)	N3'—C3'	1.416 (16)
Pd1—Br1	2.4269 (3)	C3'—N4'	1.324 (17)
Pd1—Br1^i^	2.4269 (3)	C3'—C4'	1.387 (18)
C1—N1	1.342 (3)	C4'—C5'	1.368 (19)
C1—N3	1.361 (3)	C4'—H4'	0.9500
N1—N2	1.381 (3)	C5'—C6'	1.388 (19)
N1—C8	1.465 (4)	C5'—H5'	0.9500
N2—C2	1.294 (4)	C6'—C7'	1.33 (2)
C2—N3	1.377 (4)	C6'—H6'	0.9500
C2—H2	0.9500	C7'—N4'	1.350 (18)
N3—C3	1.429 (3)	C7'—H7'	0.9500
C3—N4	1.327 (3)	C8'—C9'	1.531 (18)
C3—C4	1.384 (4)	C8'—H8'1	0.9900
C4—C5	1.374 (4)	C8'—H8'2	0.9900
C4—H4	0.9500	C9'—C10'	1.51 (2)
C5—C6	1.393 (5)	C9'—H9'1	0.9900
C5—H5	0.9500	C9'—H9'2	0.9900
C6—C7	1.334 (9)	C10'—C11'	1.52 (2)
C6—H6	0.9500	C10'—H10C	0.9900
C7—N4	1.343 (3)	C10'—H10D	0.9900
C7—H7	0.9500	C11'—H11D	0.9800
C8—C9	1.525 (4)	C11'—H11E	0.9800
C8—H8A	0.9900	C11'—H11F	0.9800
C8—H8B	0.9900	N1"—C8"	1.465 (4)
C9—C10	1.512 (5)	C8"—C9"	1.546 (13)
C9—H9A	0.9900	C8"—H8"1	0.9900
C9—H9B	0.9900	C8"—H8"2	0.9900
C10—C11	1.516 (4)	C9"—C10"	1.514 (16)
C10—H10A	0.9900	C9"—H9"1	0.9900
C10—H10B	0.9900	C9"—H9"2	0.9900
C11—H11A	0.9800	C10"—C11"	1.509 (17)
C11—H11B	0.9800	C10"—H10E	0.9900
C11—H11C	0.9800	C10"—H10F	0.9900
C1'—N3'	1.347 (17)	C11"—H11G	0.9800
C1'—N1'	1.350 (17)	C11"—H11H	0.9800
N1'—N2'	1.380 (17)	C11"—H11I	0.9800
N1'—C8'	1.457 (16)		
			
C1'^i^—Pd1—C1'	180.0 (12)	N2'—C2'—H2'	124.5
C1^i^—Pd1—C1	180.0	N3'—C2'—H2'	124.5
C1'^i^—Pd1—Br1	93.0 (11)	C1'—N3'—C2'	106.5 (17)
C1'—Pd1—Br1	87.0 (11)	C1'—N3'—C3'	126.5 (18)
C1^i^—Pd1—Br1	90.99 (8)	C2'—N3'—C3'	126.0 (18)
C1—Pd1—Br1	89.01 (8)	N4'—C3'—C4'	124.8 (17)
C1^i^—Pd1—Br1^i^	89.01 (8)	N4'—C3'—N3'	114.1 (17)
C1—Pd1—Br1^i^	90.99 (8)	C4'—C3'—N3'	120.5 (19)
Br1—Pd1—Br1^i^	180.0	C5'—C4'—C3'	119 (2)
N1—C1—N3	102.8 (2)	C5'—C4'—H4'	120.6
N1—C1—Pd1	127.85 (19)	C3'—C4'—H4'	120.6
N3—C1—Pd1	129.27 (18)	C4'—C5'—C6'	117 (2)
C1—N1—N2	114.0 (2)	C4'—C5'—H5'	121.7
C1—N1—C8	127.7 (2)	C6'—C5'—H5'	121.7
N2—N1—C8	118.3 (2)	C7'—C6'—C5'	121 (2)
C2—N2—N1	103.2 (2)	C7'—C6'—H6'	119.7
N2—C2—N3	111.3 (3)	C5'—C6'—H6'	119.7
N2—C2—H2	124.3	C6'—C7'—N4'	124 (2)
N3—C2—H2	124.3	C6'—C7'—H7'	117.8
C1—N3—C2	108.6 (2)	N4'—C7'—H7'	117.8
C1—N3—C3	127.8 (2)	C3'—N4'—C7'	114.5 (18)
C2—N3—C3	123.5 (2)	N1'—C8'—C9'	111 (2)
N4—C3—C4	125.4 (3)	N1'—C8'—H8'1	109.5
N4—C3—N3	113.9 (2)	C9'—C8'—H8'1	109.5
C4—C3—N3	120.6 (3)	N1'—C8'—H8'2	109.5
C5—C4—C3	116.6 (3)	C9'—C8'—H8'2	109.5
C5—C4—H4	121.7	H8'1—C8'—H8'2	108.1
C3—C4—H4	121.7	C10'—C9'—C8'	114 (3)
C4—C5—C6	118.8 (4)	C10'—C9'—H9'1	108.7
C4—C5—H5	120.6	C8'—C9'—H9'1	108.7
C6—C5—H5	120.6	C10'—C9'—H9'2	108.7
C7—C6—C5	119.4 (5)	C8'—C9'—H9'2	108.7
C7—C6—H6	120.3	H9'1—C9'—H9'2	107.6
C5—C6—H6	120.3	C9'—C10'—C11'	116 (3)
C6—C7—N4	124.0 (3)	C9'—C10'—H10C	108.2
C6—C7—H7	118.0	C11'—C10'—H10C	108.2
N4—C7—H7	118.0	C9'—C10'—H10D	108.2
C3—N4—C7	115.7 (2)	C11'—C10'—H10D	108.2
N1—C8—C9	111.8 (2)	H10C—C10'—H10D	107.4
N1—C8—H8A	109.3	C10'—C11'—H11D	109.5
C9—C8—H8A	109.3	C10'—C11'—H11E	109.5
N1—C8—H8B	109.3	H11D—C11'—H11E	109.5
C9—C8—H8B	109.3	C10'—C11'—H11F	109.5
H8A—C8—H8B	107.9	H11D—C11'—H11F	109.5
C10—C9—C8	113.5 (3)	H11E—C11'—H11F	109.5
C10—C9—H9A	108.9	N1"—C8"—C9"	108.7 (8)
C8—C9—H9A	108.9	N1"—C8"—H8"1	109.9
C10—C9—H9B	108.9	C9"—C8"—H8"1	109.9
C8—C9—H9B	108.9	N1"—C8"—H8"2	109.9
H9A—C9—H9B	107.7	C9"—C8"—H8"2	109.9
C9—C10—C11	113.7 (3)	H8"1—C8"—H8"2	108.3
C9—C10—H10A	108.8	C10"—C9"—C8"	110.2 (12)
C11—C10—H10A	108.8	C10"—C9"—H9"1	109.6
C9—C10—H10B	108.8	C8"—C9"—H9"1	109.6
C11—C10—H10B	108.8	C10"—C9"—H9"2	109.6
H10A—C10—H10B	107.7	C8"—C9"—H9"2	109.6
C10—C11—H11A	109.5	H9"1—C9"—H9"2	108.1
C10—C11—H11B	109.5	C11"—C10"—C9"	115.5 (17)
H11A—C11—H11B	109.5	C11"—C10"—H10E	108.4
C10—C11—H11C	109.5	C9"—C10"—H10E	108.4
H11A—C11—H11C	109.5	C11"—C10"—H10F	108.4
H11B—C11—H11C	109.5	C9"—C10"—H10F	108.4
N3'—C1'—N1'	104.3 (13)	H10E—C10"—H10F	107.5
N3'—C1'—Pd1	126.1 (14)	C10"—C11"—H11G	109.5
N1'—C1'—Pd1	129.6 (13)	C10"—C11"—H11H	109.5
C1'—N1'—N2'	112.4 (14)	H11G—C11"—H11H	109.5
C1'—N1'—C8'	126 (2)	C10"—C11"—H11I	109.5
N2'—N1'—C8'	122 (2)	H11G—C11"—H11I	109.5
C2'—N2'—N1'	102.0 (17)	H11H—C11"—H11I	109.5
N2'—C2'—N3'	110.9 (19)		
			
N3—C1—N1—N2	−0.5 (3)	Pd1—C1'—N1'—N2'	177 (3)
Pd1—C1—N1—N2	176.12 (18)	N3'—C1'—N1'—C8'	−177 (3)
N3—C1—N1—C8	179.5 (2)	Pd1—C1'—N1'—C8'	3 (5)
Pd1—C1—N1—C8	−4.0 (4)	C1'—N1'—N2'—C2'	13 (5)
C1—N1—N2—C2	0.8 (3)	C8'—N1'—N2'—C2'	−172 (4)
C8—N1—N2—C2	−179.1 (3)	N1'—N2'—C2'—N3'	−19 (7)
N1—N2—C2—N3	−0.8 (4)	N1'—C1'—N3'—C2'	−9 (5)
N1—C1—N3—C2	−0.1 (3)	Pd1—C1'—N3'—C2'	171 (4)
Pd1—C1—N3—C2	−176.6 (3)	N1'—C1'—N3'—C3'	−179 (3)
N1—C1—N3—C3	−176.9 (2)	Pd1—C1'—N3'—C3'	2 (5)
Pd1—C1—N3—C3	6.6 (4)	N2'—C2'—N3'—C1'	19 (7)
N2—C2—N3—C1	0.6 (4)	N2'—C2'—N3'—C3'	−172 (4)
N2—C2—N3—C3	177.6 (3)	C1'—N3'—C3'—N4'	136 (3)
C1—N3—C3—N4	−150.6 (2)	C2'—N3'—C3'—N4'	−31 (6)
C2—N3—C3—N4	33.1 (4)	C1'—N3'—C3'—C4'	−51 (4)
C1—N3—C3—C4	33.2 (4)	C2'—N3'—C3'—C4'	142 (5)
C2—N3—C3—C4	−143.2 (3)	N4'—C3'—C4'—C5'	−3 (2)
N4—C3—C4—C5	1.6 (4)	N3'—C3'—C4'—C5'	−175 (3)
N3—C3—C4—C5	177.3 (3)	C3'—C4'—C5'—C6'	−1 (2)
C3—C4—C5—C6	−0.8 (5)	C4'—C5'—C6'—C7'	0 (4)
C4—C5—C6—C7	0.1 (7)	C5'—C6'—C7'—N4'	4 (5)
C5—C6—C7—N4	0.2 (7)	C4'—C3'—N4'—C7'	7 (4)
C4—C3—N4—C7	−1.3 (4)	N3'—C3'—N4'—C7'	179 (3)
N3—C3—N4—C7	−177.3 (2)	C6'—C7'—N4'—C3'	−7 (5)
C6—C7—N4—C3	0.4 (5)	C1'—N1'—C8'—C9'	−95 (4)
C1—N1—C8—C9	123.8 (3)	N2'—N1'—C8'—C9'	91 (3)
N2—N1—C8—C9	−56.3 (3)	N1'—C8'—C9'—C10'	−105 (5)
N1—C8—C9—C10	−63.5 (4)	C8'—C9'—C10'—C11'	144 (6)
C8—C9—C10—C11	176.6 (3)	N1"—C8"—C9"—C10"	72.0 (15)
N3'—C1'—N1'—N2'	−2 (4)	C8"—C9"—C10"—C11"	173.7 (19)

Symmetry code: (i) −*x*+1, −*y*+1, −*z*+1.
